# An Archaeal Cyclodextrin Glycosyltransferase from *Haloferax* sp.: Characterization and Application in Starch Degradation

**DOI:** 10.3390/ijms27125532

**Published:** 2026-06-18

**Authors:** Yan Li, Anan Li, Xue Long, Yuqing Cao, Aiyue Zhang, Jingang Gu, Rui Ma, Guogang Zhao

**Affiliations:** 1 College of Life Sciences, Hebei Agricultural University, Baoding 071001, China; liyan010506@163.com (Y.L.); ananli2020@163.com (A.L.); longxue_2020@163.com (X.L.); cyq_bc2024@126.com (Y.C.); zayzay2002@163.com (A.Z.); 2 Key Laboratory of Microbial Resources Collection and Preservation, Institute of Agricultural Resources and Regional Planning, Chinese Academy of Agricultural Sciences, Beijing 100086, China; gujingang@caas.cn

**Keywords:** cyclodextrins (CDs), cyclodextrin glycosyltransferase (CGTase), *Haloferax* sp., halophilic archaeon, starch conversion, thermostable enzyme

## Abstract

Cyclodextrin glycosyltransferase (CGTase) is a highly valuable biocatalyst in industrial starch conversion, particularly for the synthesis of cyclic oligosaccharides. In this study, a CGTase, designated *Hf*CGT, was cloned from *Haloferax* sp. and heterologously expressed in *Escherichia coli*. The recombinant enzyme was purified and biochemically characterized. *Hf*CGT exhibited maximal catalytic activity at 70 °C and pH 8.0, tolerance to metal ions and EDTA, and enhanced activity in the presence of 1 M NaCl and Ca^2+^. High-performance liquid chromatography (HPLC) and high-resolution mass spectrometry (HRMS) analyses revealed that the starch products by *Hf*CGT degradation were mainly large-ring cyclodextrins (LR-CDs) with polymerization degrees of 9 to 20. Altogether, the thermostability, haloalkaliphilic, and distinctive product profile make *Hf*CGT a promising biocatalyst for pharmaceutical, food, and biotechnological applications.

## 1. Introduction

Cyclodextrins (CDs) are cyclic oligosaccharides that consist of six to eight glucose units linked by α-(1,4)-glycosidic bonds. These molecules possess a hydrophilic exterior and a relatively hydrophobic internal cavity, and this unique structural configuration enables them to encapsulate various compounds and modify their physicochemical properties. Thus, CDs have been widely applied in the fields of food, pharmaceuticals, cosmetics, environmental protection, and chemicals [1,2,3,4,5,6]. In contrast, large-ring cyclodextrins (LR-CDs) are cyclic glucans composed of nine or more glucose units [7]. Compared with conventional CDs, LR-CDs exhibit higher aqueous solubility, a larger hydrophobic cavity, and a unique flexible structure, which confer considerable application potential across multiple fields. In the pharmaceutical industry, LR-CDs have been used to improve the physicochemical properties of drugs, including stability, solubility, and bioavailability [8,9,10]. In the food industry, LR-CDs are used to enhance flavor and texture, inhibit starch retrogradation, and prevent oxidative discoloration of fruit juices [11,12]. In biological systems, LR-CDs have also been used as artificial molecular chaperones that facilitate the correct refolding of denatured proteins, thereby restoring enzymatic activity [13]. Thus, LR-CDs attract much interest from industries due to their high values and versatile uses.

Cyclodextrin glycosyltransferase (CGTase, EC 2.4.1.19), belonging to the glycoside hydrolase family 13 (GH 13), is a key enzyme for catalyzing reversible intermolecular and intramolecular α-1,4-transglycosylation reactions of starch [14,15]. Through these reactions, CGTase is capable of producing various CDs and LR-CDs, and has attracted considerable interest in applied research. To date, CGTases have been predominantly identified from bacterial sources, particularly the genus *Bacillus*. In contrast, only a limited number of archaeal CGTases have been reported, including those from the thermophilic *Thermococcus* sp. strain B1001, *Thermococcus kodakaraensis* KOD1, and *Pyrococcus furiosus* DSM3638, as well as the halophilic *Haloferax mediterranei*. Most microbial CGTases, including the known archaeal copartners, initially produce LR-CDs, which decrease progressively along with the reaction and release α/β/γ-CDs as the final products [11,16,17,18,19]. Thus, LR-CDs are difficult to obtain by enzymatic conversion of starch.

Halophilic archaea are capable of adapting to extreme environments, such as high temperature, high salinity, and extreme pHs. Among them, strains of the genus *Haloferax* grow well over wide salt concentration ([Na^+^] = 1.0–5.2 M) and temperature ranges (15–55 °C) [20]. In addition, archaeal enzymes have unique advantages in reduced risk of microbial contamination and improved solubility of substrates and products for industrial starch conversion under stringent conditions. The extraordinary stability of archaeal enzymes arises from the combined effects of multiple factors, including an increased number of surface acidic residues, strengthened ion pair networks, and the formation of hydration layers. These mechanisms lay a foundation for subsequent protein engineering to further improve the robustness of enzymes in industrial applications [21,22]. In this study, we identified and characterized an archaeal CGTase from *Haloferax* sp. EPS crtB. The enzyme showed distinguished catalytic properties, stability under high salinity and alkaline conditions, as well as the ability to synthesize LR-CDs. These excellent properties make it promising as a biocatalyst in various industries.

## 2. Results

### 2.1. Sequence Analysis of the HfCGT

**Figure 1:**
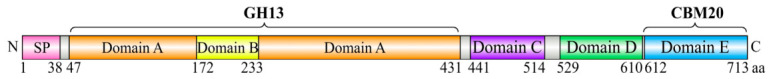
The schematic representation of the structure elements of deduced *Hf*CGT.

The gene *Hf*CGT is 2 142 bp in length, and codes for a polypeptide of 713 amino acids with a molecular weight of 78 599.44 Da. The theoretical isoelectric point was predicted by ProtParam is 4.19. According to the Carbohydrate-Active enZYmes (CAZy) database, *Hf*CGT is classified into the glycoside hydrolase family 13 (GH13). Further predictions with SignalP 5.0 and CD-Search indicated that deduced *Hf*CGT consists of a signal peptide of 38 residues, an N-terminal catalytic domain of GH13, a linker peptide, and a C-terminal starch-binding domain of carbohydrate-binding module family 20 (CBM20) (Figure 1).

#### 2.1.1. Three-Dimensional Modeling and Multiple Sequence Alignment

**Figure 2:**
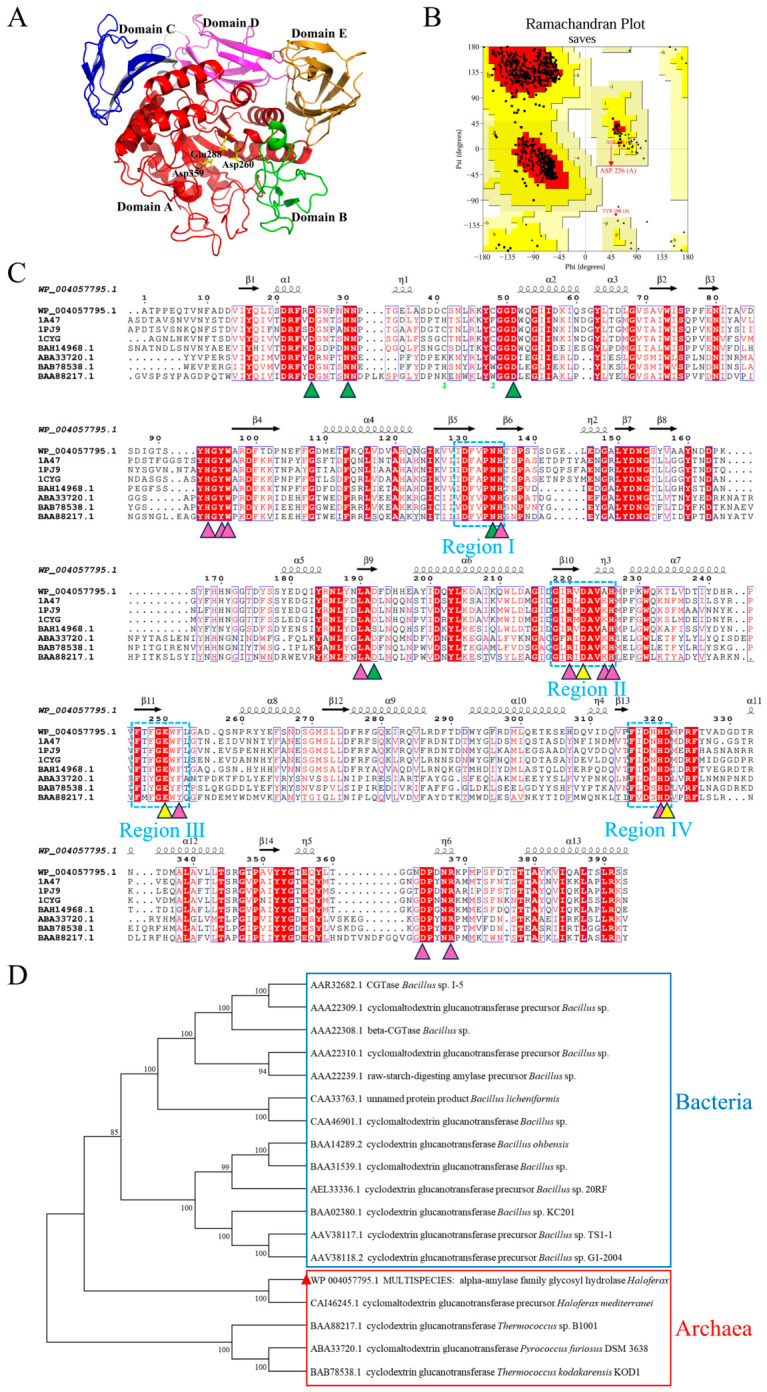
Sequence analysis of *Hf*CGT. (**A**) The three-dimensional structure of modeled *Hf*CGT with putative catalytic residues Asp260, Glu288, and Asp359, marked in yellow; (**B**) Ramachandran plot; (**C**) multiple sequence alignment of *Hf*CGT and the CGTases from *Thermoanaerobacterium thermosulfurigenes* (PDB ID: 1A47), *Bacillus circulans* (PDB ID: 1PJ9), *Geobacillus stearothermophilus* (PDB ID: 1CYG), *Evansella clarkii* (GenBank Accession No.: BAH14968.1), *Haloferax mediterranei* (GenBank Accession No.: CAI46245.1), *Pyrococcus furiosus* DSM 3638 (GenBank Accession No.: ABA33720.1), *Thermococcus kodakarensis* KOD1 (GenBank Accession No.: BAB78538.1), and *Thermococcus* sp. B1001 (GenBank Accession No.: BAA88217.1). The characteristic motifs (blue boxes), the catalytic residues (yellow triangles), the active sites (pink triangles), and the Ca^2+^ binding sites (green triangles) are indicated; (**D**) phylogenetic analyses of *Hf*CGT and known counterparts of GH13. The red triangle represents *Hf*CGT.

As shown in Figure 2A, the three-dimensional structure of *Hf*CGT was modeled and visualized using the AlphaFold3 (v 3.0.1) and PyMOL (v 3.1.3) software. The overall average of the predicted Local Distance Difference Test (pLDDT) score was 96.5, indicating a high-confidence prediction. Additionally, the model quality was assessed using the SAVES v6.1 online platform. As presented in Figure 2B, the Ramachandran plot showed that 91.4% of the amino acid residues were located in the most favored regions, which is higher than the generally accepted threshold for structural reliability (>90%), indicating that the predicted three-dimensional structure model is conformationally reasonable and can be used for subsequent structural feature analysis. *Hf*CGT consists of five distinct domains (A–E) and contains four conserved α-amylase characteristic motifs (Regions I–IV) in its amino acid sequence, located at ^167^IDFVPNH^173^, ^256^GIRVDAVAH^264^, ^284^FTFGEWFL^291^, and ^354^FIDNHD^359^, respectively. The catalytic domain of *Hf*CGT is a typical (β/α)_8_ barrel containing two aspartic acid and one glutamic acid residue (i.e., Asp260, Asp359, and Glu288) as the catalytic residues.

Multiple sequence alignment of *Hf*CGT and other reported homologues revealed that the catalytic residues and four conserved amino acid sequences are highly conserved across the known CGTases (Figure 2C). The active site and calcium ion-binding site also exhibit a high degree of conservation. Notably, *Hf*CGT shares 99.16% amino acid sequence identity with the CGTase from the archaeon *Haloferax mediterranei*, suggesting that they may share a common evolutionary ancestor and potentially possess similar catalytic properties. *Hf*CGT also shows high identity (59.73%) with the CGTase from *Geobacillus stearothermophilus*, but exhibits low sequence similarities (32–38%) to other archaeal CGTases in the SwissPro database, which may indicate potential divergence in functional characteristics among these enzymes.

#### 2.1.2. Phylogenetic Analysis

To further elucidate the evolutionary relationship between *Hf*CGT and other GH13 CGTases of different sources, a phylogenetic tree was constructed based on protein sequences using the Neighbor-Joining (NJ) method. As shown in Figure 2D, the CGTases under study cluster into two distinct clades: Clade I contains CGTases of bacterial origin, while Clade II includes those from archaea. *Hf*CGT and the archaeal enzyme belong to the same clade and are most closely related to the CGTase from *Haloferax mediterranei*; they may share similar mechanisms of halophilic adaptation.

### 2.2. Expression and Purification of Recombinant HfCGT

**Figure 3:**
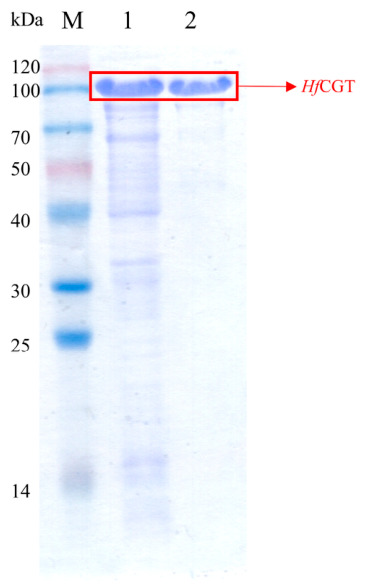
SDS-PAGE analysis of the purified *Hf*CGT. Lane M: Protein molecular weight marker; Lane 1: crude cell extract after IPTG induction; Lane 2: purified *Hf*CGT after Ni-NTA affinity chromatography.

Recombinant *Hf*CGT was expressed in soluble form in *E*. *coli* BL21 (DE3). Induction at 30 °C for 10 h was then used in subsequent experiments (Figure S1). Through nickel affinity chromatography, the recombinant *Hf*CGT was purified to apparent homogeneity with a purification fold of 6.85 and a recovery rate of 48.4%, respectively (Table S1). SDS-PAGE analysis, using a 12% separating gel, demonstrated that the apparent molecular weight of the purified protein was 90.2 kDa (Figure 3), which was consistent with the theoretical molecular weight of the mature *Hf*CGT protein fused with an N-terminal His_6_-tag, a C-terminal His_6_-tag, and a SUMO-tag.

### 2.3. Biochemical Characterization of Recombinant HfCGT

**Figure 4:**
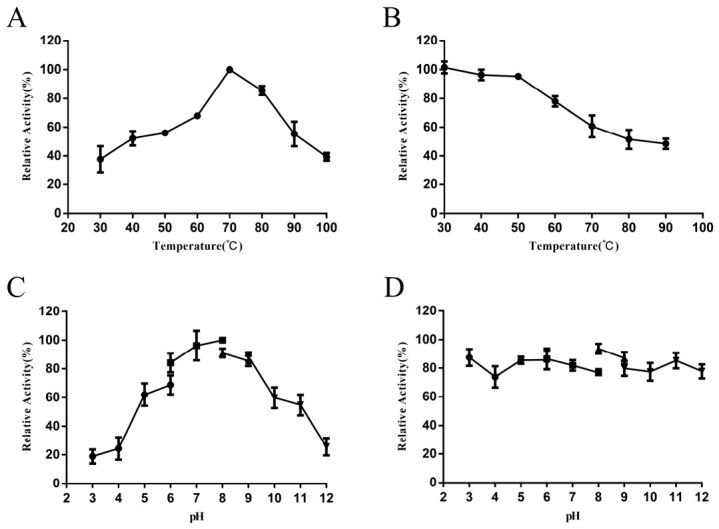
Enzymatic properties of purified recombinant *Hf*CGT: (**A**) optimal temperature; (**B**) thermal stability; (**C**) optimal pH; (**D**) pH stability. (●) citrate-Na_2_HPO_4_ buffer (pH 3.0–6.0); (■) NaH_2_PO_4_-Na_2_HPO_4_ buffer (pH 6.0–8.0); (▲) tris-HCl buffer (pH 8.0–9.0); (▼) Gly-NaOH buffer (pH 9.0–12.0). Error bars represent SD (*n* = 3).

The purified recombinant *Hf*CGT exhibited high activity within the temperature range of 60–80 °C, with maximum activity detected at 70 °C (Figure 4A). The highest activity of the purified recombinant *Hf*CGT was observed at pH 8.0 (Figure 4B). The enzyme showed excellent thermal stability, retaining over 90% of its initial activity after incubation at 30–60 °C for 1 h, and more than 50% of its initial activity even after incubation at 70–80 °C for 1 h (Figure 4C). Moreover, *Hf*CGT maintained stability over a broad pH range, with more than 80% of its enzymatic activity after incubation at pH 5.0–12.0 for 1 h (Figure 4D).

The effects of various metal ions and chemical reagents on *Hf*CGT activity were determined under optimal conditions (Table 1). *Hf*CGT was activated in the presence of Na^+^, Ca^2+^, and K^+^, and showed tolerance to Mg^2+^, Mn^2+^, and EDTA. Partial inhibition of *Hf*CGT activity was observed in the presence of Fe^2+^, Fe^3+^, Zn^2+^, and Ni^2+^, whereas Al^3+^, Cu^2+^, Ag^+^, Hg^2+^, SDS, and β-mercaptoethanol completely inhibited *Hf*CGT activity.

**Figure 5:**
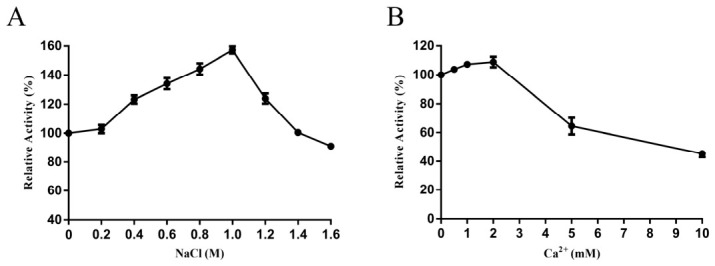
Enhanced activity of *Hf*CGT in the presence of NaCl (**A**) and CaCl_2_ (**B**). Error bars represent SD (*n* = 3).

Given that *Haloferax* sp. EPS crtB belongs to the halophilic archaea, the salt tolerance of *Hf*CGT under varying concentrations of Na^+^ was also determined. Moreover, considering Ca^2+^ as a classical stabilizing cofactor for CGTase, the effect of Ca^2+^ on *Hf*CGT activity was also evaluated. Using 1% (*w*/*v*) soluble starch as the substrate, *Hf*CGT achieved the highest activity in the presence of 1 M NaCl (Figure 5A), and the activity was enhanced by 12% when the Ca^2+^ concentration was set to 2 mM (Figure 5B).

### 2.4. Substrate Specificity and Kinetics

To identify the substrate specificity of *Hf*CGT, soluble starch, amylose, and amylopectin were selected as the substrates. *Hf*CGT exhibited the highest activity towards amylose (36.78 ± 1.88 U/mg), moderate on soluble starch (28.16 ± 2.97 U/mg), and weak on amylopectin (3.06 ± 3.76 U/mg), respectively.

The kinetic parameters, *V*ₘₐₓ, *K*ₘ, *K*_cat_, and *K*_cat_/*K*ₘ, of *Hf*CGT towards soluble starch were determined to be 28.91 ± 7.61 μmol/min/mL, 0.344 ± 1.03 mg/mL, 300.73 ± 2.83 s^−1^, and 847.13 ± 6.97 s^−1^·mL/mg, respectively.

### 2.5. Analysis of the Hydrolytic Products

**Figure 6:**
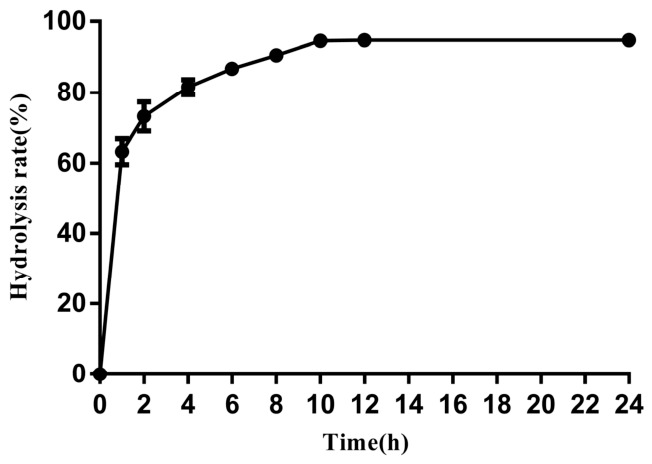
Time-course of soluble starch hydrolysis by *Hf*CGT. Error bars represent SD (*n* = 3).

The hydrolysis process of soluble starch by *Hf*CGT is shown in Figure 6. Within 0–10 h, the hydrolysis rate kept increasing, reaching the maximum conversion rate of 94.82% at 12 h. After that, the substrate was completely hydrolyzed, and the reaction reached equilibrium.

**Figure 7:**
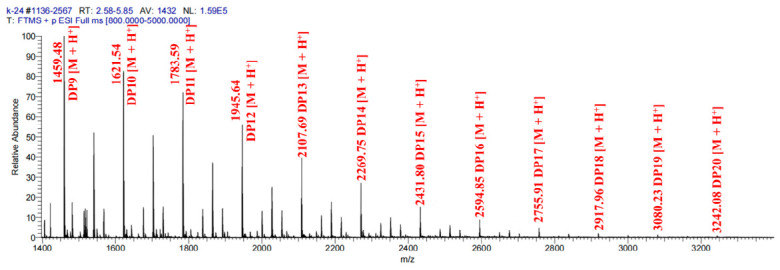
HRMS spectra of LR-CD products. Major peaks correspond to [M + Na]^+^ of LR-CDs with DP 9–20. The *m/z* to DP assignments are detailed in Supplementary Table S2.

The high-performance liquid chromatography (HPLC) results indicated that no α/β/γ-CD was formed during the starch degradation by *Hf*CGT. Further product analysis with high-resolution mass spectrometry (HRMS) indicated that the dominant hydrolysis products of *Hf*CGT were primarily LR-CDs of DP 9–20 (Figure 7 and Supplementary Table S2).

To highlight the unique product profile of *Hf*CGT, its catalytic characteristics were compared with those of representative CGTases derived from bacteria and archaea (Table 2). The wild-type *Hf*CGT characterized in this study preferentially produces LR-CDs with DP 9 to 20 under optimal conditions, while maintaining high thermostability and excellent tolerance to high-salt and alkaline environments. These distinct features endow this archaeal CGTase with unique advantages for industrial starch conversion applications.

## 3. Discussion

In this study, a CGTase derived from the haloalkaliphilic archaeon *Haloferax* sp. EPS crtB was successfully cloned and heterologously expressed in *E. coli*. Sequence analysis revealed that *Hf*CGT consists of five distinct domains (A–E) and three catalytic residues, which are highly conserved in CGTases of GH13 [25]. The catalytic domain A adopts a typical (β/α)_8_-barrel fold with four conserved α-amylase motifs, with Asp260, Glu288, and Asp359 serving as essential catalytic residues. Similar to other known crystal structures of CGTases, a short domain B is located within domain A [26,27,28,29]. Domains B and C contribute to the structural stability of the catalytic domain and substrate-binding capacity [30,31], which accounts for the ability of *Hf*CGT to remain active under varying temperature and pH conditions. Domain E belongs to the CBM20 that contains two starch-binding sites, thus facilitating the enzyme–substrate interactions by disrupting the surface structure of starch and enhancing the catalytic efficiency of domain A [32,33,34]. In contrast, the specific function of domain D remains unclear. It may encode unique structural elements associated with the salt tolerance of the enzyme. In future studies, site-directed mutagenesis, molecular docking, and molecular dynamics simulations can be applied to verify this hypothesis.

Comprehensive enzymatic characterization revealed that *Hf*CGT exhibits an optimal catalytic temperature of 70 °C and an optimal pH of 8.0. Most CGTases from *Bacillus* spp. exhibit optimal performance at moderate temperatures (40–60 °C) and a near-neutral pH, consistent with mesophilic growth conditions [29,35]. Although certain bacterial CGTases retain partial activity under alkaline conditions, their stability and catalytic efficiency are often compromised when exposed to elevated temperatures or prolonged alkaline stress [23,36]. Furthermore, *Hf*CGT exhibited maximum activity in the presence of 1 M NaCl and maintained good stability over a broad pH range, demonstrating halophilic characteristics consistent with the living environment of halophilic archaea. Similarly, the CGTase from *Haloferax mediterranei* exhibited optimal activity at 1.5 M NaCl, suggesting that halophilic archaeal CGTases may share common mechanisms of salt adaptation [19]. The abundance of acidic residues on the protein surface likely promotes cation binding and hydration layer formation, thereby helping prevent dehydration and denaturation under high-salt conditions. Although *Hf*CGT has a lower optimal temperature than CGTases from hyperthermophilic archaea such as *Thermococcus kodakaraensis* KOD1 and *Pyrococcus furiosus* DSM3638 [17,18], it exhibits superior tolerance to high salinity and alkaline conditions. Recent enzyme engineering strategies have focused on enhancing thermostability and halotolerance through rational design and directed evolution [37,38]. The inherent stability of *Hf*CGT under high-salt and alkaline conditions renders further engineering unnecessary, highlighting the great potential of exploring extremophilic microorganisms for the direct development of efficient biocatalysts.

In addition to its adaptation to alkaline and moderate to high temperature conditions, *Hf*CGT exhibits a distinctive product profile. Most previously reported CGTases predominantly produce α-, β-, and γ-CDs, with β-CD frequently being the major product. Some researchers have found that LR-CDs were abundant in the early stages of starch degradation reactions, but as the reaction time extended, they were ultimately degraded into regular CDs, making it difficult to stably accumulate [39,40]. To obtain LR-CDs, researchers typically need to engineer CGTases through laborious and time-consuming methods. Sonnendecker et al. achieved the specific synthesis of LR-CDs with a degree of DP 8–12 through multiple site-directed mutations of CGTase from *Bacillus* sp. G-825-6 [41]. Kaulpiboon et al. used a molecular imprinting method with a DP 12 LR-CD as a template to treat CGTase from *Paenibacillus* sp. A11, thereby achieving a high yield of LR-CDs (DP 12) [42]. In both cases, extensive protein engineering or template-assisted manipulation was required. In this study, *Hf*CGT primarily produced LR-CDs with DP 9–20 after degrading soluble starch, and no major common CDs were detected even after 24 h of reaction. Notably, as a wild-type CGTase derived from archaea, *Hf*CGT can stably produce LR-CDs without any protein engineering modifications, which is extremely rare among the currently reported GH13 family CGTases. This natural ability positions *Hf*CGT as a rare and valuable biocatalyst. However, the final product of starch degradation by *H. mediterranei* CGTase, which shares a high sequence similarity with *Hf*CGT, was β-CD. Sequence alignment revealed differences at amino acid positions 433–438 between the two enzymes, suggesting that these six amino acid residues may influence the product specificity of the enzyme during starch degradation. Further site-directed mutagenesis and molecular dynamics simulations will be performed to verify the proposed structure–function relationship. Previous structure–function studies have demonstrated that CGTase hydrolysis specificity is primarily governed by the overall architecture of the active site, including the configuration of the substrate-binding groove and surrounding flexible regions, rather than by a single catalytic residue [43,44,45]. The production of LR-CDs may be related to structural features that favor the binding and stabilization of longer glucan chains during cyclization.

In conclusion, an archaeal CGTase from *Haloferax* sp. EPS crtB was successfully cloned and heterologously expressed in *E*. *coli* BL21(DE3). The recombinant enzyme, *Hf*CGT, exhibited high thermostability and pH stability under moderate to high temperature ranges and alkaline conditions. Notably, *Hf*CGT displayed a distinct CD product profile, preferentially producing LR-CDs under optimal conditions. Structural factors governing the product specificity of CGTases include subsite architectures and key amino acid residues [46]. On this basis, AI-driven enzyme engineering is expected to further improve the LR-CD yield, catalytic efficiency, and operational stability of *Hf*CGT during industrial starch conversion [47]. Although further studies are required to elucidate the underlying catalytic mechanism, these findings expand the current knowledge of CGTase diversity and provide a basis for future structure–function investigations and biosynthetic studies of LR-CDs.

## 4. Materials and Methods

### 4.1. Chemicals and Strains

*E. coli* DH5α and *E. coli* BL21(DE3) (BioMed, Beijing, China) were used as host strains for DNA manipulation and heterologous expression, respectively. Soluble starch, amylose, and amylopectin were purchased from Yuanye Bio-Technology Co., Ltd. (Shanghai, China). All chemicals used were of analytical grade and commercially available.

### 4.2. Sequence Analysis and Synthesis

The gene sequence of *Hf*CGT (NCBI Reference Sequence: WP_004057795.1) was retrieved from the NCBI database. Putative signal peptide as well as the module structure were predicted using SignalP 5.0 (https://services.healthtech.dtu.dk/services/SignalP-5.0/, accessed on 8 February 2026) and a simple module structure study tool (https://www.ncbi.nlm.nih.gov/Structure/cdd/wrpsb.cgi, accessed on 8 February 2026). At the same time, the predicted protein conformations were analyzed using the SAVES v6.1 online platform (https://saves.mbi.ucla.edu/) to assess their reasonableness. Subsequently, the *Hf*CGT sequence without the signal peptide was synthesized after codon optimization by GenScript Biotech Co., Ltd. (Nanjing, China), and flanked with *Bam*H I and *Hin*d III restriction sites. The target fragment was cloned into the pET-28a-SUMO vector. The resulting recombinant plasmid *Hfcgt*-pET-28a-SUMO was verified by Sanger sequencing.

### 4.3. Homology Modeling and Multiple Sequence Alignment

Homology modeling of *Hf*CGT was performed using AlphaFold3 (https://alphafoldserver.com), followed by visualization analysis via PyMOL software, with the key residues of *Hf*CGT annotated. Sequence alignment of *Hf*CGT and close homologues was performed using ClustalW (https://www.genome.jp/tools-bin/clustalw, accessed on 2 April 2026), and the alignment results were color-coded using ESPript 3.2 (https://espript.ibcp.fr/ESPript/ESPript/, accessed on 2 April 2026). A phylogenetic tree of *Hf*CGT and other GH13 counterparts was constructed using MEGA 11.0 software with the Neighbor-Joining method. The Poisson model was used to calculate evolutionary distances, and a bootstrap analysis was performed with 1000 replicates.

### 4.4. Expression and Purification of the Recombinant HfCGT

The recombinant plasmid *Hfcgt*-pET-28a-SUMO was transformed into *E. coli* BL21 (DE3). Transformants were grown in LB medium containing 100 μg/mL of kanamycin at 37 °C with shaking. When 600 nm (OD_600_) reached 0.6–0.8, protein expression was induced with 0.1 mM IPTG at 20 °C for 22 h, 25 °C for 16 h, 30 °C for 10 h, and 37 °C for 6 h, respectively.

*Hf*CGT was purified by nickel affinity chromatography (TransGen Biotech, Beijing, China). After induction of *Hf*CGT at 30 °C for 10 h, cells were harvested by centrifugation and resuspended in Ni-NTA equilibration buffer (50 mM NaH_2_PO_4_ containing 10 mM imidazole and 300 mM NaCl, pH 8.0). Cell disruption was performed with a 3 s sonication pulse, 5 s interval, 30% power, and a total disruption time of 30 min. The crude enzyme was collected by centrifuging at 12,000 rpm and 4 °C for 10 min to remove insoluble cell debris, followed by loading onto a Ni-NTA agarose column. The target *Hf*CGT was obtained with an elution buffer (50 mM NaH_2_PO_4_ containing 350 mM imidazole and 300 mM NaCl, pH 8.0). Following desalination using an Amicon Ultra-30K centrifugal filter unit (Millipore, Billerica, MA, USA), *Hf*CGT was concentrated and stored in 50 mM NaH_2_PO_4_-Na_2_HPO_4_ buffer (pH 8.0) at −20 °C.

Sodium dodecyl sulfate-polyacrylamide gel electrophoresis (SDS-PAGE) was performed with a 12% separating gel. Using bovine serum albumin (BSA) as the standard, the concentration of the target protein *Hf*CGT was determined by the Bradford method.

### 4.5. Enzyme Activity Assay

The reaction mixture contained 0.5–1 μg of purified *Hf*CGT, 200 μL of 1% (*w*/*v*) soluble starch, and 200 μL of 50 mM NaH_2_PO_4_-Na_2_HPO_4_ (pH 8.0). After incubation at 70 °C for 10 min, the reaction was stopped with 0.5 mL of 0.5 M acetic acid, followed by the addition of 3 mL of 0.005% (*w*/*v*) iodine solution. Absorbance was measured at 700 nm. One unit of activity was defined as the amount of enzyme causing a 10% reduction in absorbance under these conditions.

### 4.6. Biochemical Characterization

Optimal temperature and pH: An optimal temperature was determined as 30–90 °C at pH 8.0. Optimal pH was tested at 70 °C using citrate-Na_2_HPO_4_ buffer (pH 3.0–6.0), NaH_2_PO_4_-Na_2_HPO_4_ buffer (pH 6.0–8.0), Tris-HCl buffer (pH 8.0–9.0), and Gly-NaOH buffer (pH 9.0–12.0). Activity under optimal conditions was set as 100%.

Thermal and pH stability: For thermal stability, the enzyme was incubated at 30–90 °C for 1 h prior to activity measurement. For pH stability, the enzyme was incubated in buffers with pHs of 3.0–12.0 for 1 h before assaying residual activity. The untreated enzyme served as the control (100%).

Metal ions and reagents: The enzyme was assayed at 70 °C and pH 8.0 with 1 mM of various metal ions or reagents (NaCl, KCl, MgCl_2_, ZnCl_2_, FeCl_2_, CaCl_2_, AlCl_3_, CuSO_4_, NiCl_2_, MnCl_2_, FeCl_3_, HgCl_2_, AgNO_3_, EDTA, SDS, and β-mercaptoethanol). Activity without additives was defined as 100%.

Effects of Na^+^ and Ca^2+^ on *Hf*CGT activity: Using 1% soluble starch as the substrate, reactions were performed at 70 °C, pH 8.0, using NaCl (0–1.6 M) or CaCl_2_ (0–10 mM). Activity was measured under standard conditions.

All experiments were repeated three times. The results of multiple experiments are reported as means ± standard deviation (SD). A *p*-value less than 0.05 was considered statistically significant.

### 4.7. Substrate Specificity

The substrate specificity of *Hf*CGT was evaluated using 1% (*w*/*v*) of soluble starch, amylose, and amylopectin as the substrates. Enzyme activity was assayed under standard conditions, absorbance (A) was measured at 700 nm, and the specific activities of *Hf*CGT for degrading the three substrates were calculated, respectively. All experiments were repeated three times. The results of multiple experiments are reported as means ± SD.

### 4.8. Kinetic Analysis

The kinetic parameters of *Hf*CGT were determined using soluble starch as the substrate at final concentrations ranging from 0.5 to 2.0 mg/mL in 50 mM NaH_2_PO_4_-Na_2_HPO_4_ buffer (pH 8.0) containing 2 mM Ca^2+^ under standard assay conditions. GraphPad Prism 5 software was used for non-linear regression analysis to fit the Michaelis–Menten equation, and kinetic parameters, including *K*ₘ, *V*ₘₐₓ, *K*_cat_, and *K*_cat_/*K*ₘ, were calculated. All experiments were repeated three times. The results of multiple experiments are reported as means ± SD.

### 4.9. Determination of Hydrolytic Products

The hydrolytic activity of *Hf*CGT was determined using soluble starch as the substrate. The reaction system containing 1 mg/mL of *Hf*CGT, 1% soluble starch in 50 mM NaH_2_PO_4_-Na_2_HPO_4_ buffer (pH 8.0), and 2 mM Ca^2+^ was incubated at 70 °C for 24 h. Samples were collected at different time intervals. The reaction was terminated by adding 0.5 M acetic acid, followed by 0.005% (*w*/*v*) iodine solution for color development. Absorbance was measured at a wavelength of 700 nm.

Hydrolysis products were analyzed using the Agilent 1260 Series High-Performance Liquid Chromatography (HPLC) system equipped with a refractive index detector (RID). Separation was achieved on a COSMOSIL Sugar-D column (4.6 mm I.D. × 250 mm). The mobile phase consisted of acetonitrile/water (65:35, *v*/*v*) at a flow rate of 1 mL/min. The injection volume was 10 μL, and the column temperature was maintained at 30 °C.

High-resolution mass spectrometry (HRMS) analysis was performed using a Q Exactive Plus mass spectrometer (Thermo Fisher Scientific, Waltham, MA, USA) equipped with an electrospray ionization (ESI) source operating in positive ion mode. The mobile phases consisted of 0.1% (*v*/*v*) formic acid in water (A) and 0.1% (*v*/*v*) formic acid in acetonitrile (B). The elution program was as follows: 0–1.5 min, 99% A; 1.5–15 min, 1–99% B; 15–20 min, 99% B; 20–21 min, 99–1% B; and 21–30 min, 99% A for column re-equilibration. The flow rate was set at 0.3 mL/min [48]. The spray voltage was set to 3.5 kV, the capillary temperature was 320 °C, and the heater temperature was 350 °C. Sheath gas and auxiliary gas were set to 35 and 10 arbitrary units, respectively. Full-scan mass spectra were acquired over an *m/z* range of 800–5000, enabling the detection of LR-CDs. Data acquisition and analysis were performed using Xcalibur (v 4.5.474.0) software (Thermo Fisher Scientific, Waltham, MA, USA).

## Figures and Tables

**Table 1:** Effects of metal ions and chemical reagents on *Hf*CGT activity.

Chemicals	Relative Activity (%)
NaCl	134.38 ± 6.56
CaCl_2_	127.34 ± 3.91
KCl	123.44 ± 7.81
MgCl_2_	96.09 ± 7.16
MnCl_2_	91.41 ± 5.47
FeCl_2_	50.0 ± 6.58
FeCl_3_	46.09 ± 7.32
ZnCl_2_	44.53 ± 8.59
NiCl_2_	41.41 ± 4.22
AlCl_3_	N.D.
CuSO_4_	N.D.
AgNO_3_	N.D.
HgCl_2_	N.D.
EDTA	102.13 ± 3.13
SDS	N.D.
β-Mercaotoethanol	N.D.

N.D. indicates no detectable activity.

**Table 2:** Comparison of the properties of microbial and archaeal CGTases.

Microbial Source	Optimum pH	Optimum Temperature (°C)	pH Stability	Thermal Stability (°C)	*K_m_* (mg/mL)	Predominant Product	Conversion Rate (%)	Reference
**Bacteria**								
*Amphibacillus* sp.	8.0	50	5.0–11.0	45–75	1.70	β-CD	67.2	[2]
*Bacillus pseudalcaliphilus*	6.0/9.0	60	5.0–10.0	60–70	–	β-CD	47.0	[23]
*Bacillus megaterium*	7.2	60	6.0–10.5	30	3.40	β-CD	49.0	[6]
*Gracilibacillus alcaliphilus*	7.0	60	6.0–9.0	30–50	2.06	β-CD	37.7	[24]
**Archaea**								
*Thermococcus kodakaraensis*	5.5–6.0	80	–	85 (+ Ca^2+^)	–	β-CD	–	[17]
*Pyrococcus furiosus*	5.0	95	–	100	–	β-CD	–	[18]
*Haloferax* sp. EPS crtB	8.0	70	5.0–12.0	30–80	0.34	LR-CDs (DP 9–20)	94.8	This study

## Data Availability

The original contributions presented in this study are included in the article. Further inquiries can be directed to the corresponding authors.

## Supplementary Materials

The following supporting information can be downloaded at: https://www.mdpi.com/article/10.3390/ijms27125532/s1.

## Abbreviations

The following abbreviations are used in this manuscript:

CGTase	Cyclodextrin glucosyltransferase
CDs	Cyclodextrins
LR-CDs	Large-ring cyclodextrins
DP	Degree of polymerization
SBD	Starch-binding domain
I.D.	Inner diameter
