# Supplementary material for: An Archaeal Cyclodextrin Glycosyltransferase from Haloferax sp.: Characterization and Application in Starch Degradation
Source: Int J Mol Sci. 2026 Jun 18;27(12):5532. doi: 10.3390/ijms27125532 (PMC13299525; doi:10.3390/ijms27125532)
Supplement: Supplementary file 1 [file ijms-27-05532-s001.zip › ijms-4333057-supplementary.pdf]

Supplementary information

An archaeal cyclodextrin glycosyltransferase from *Haloferax* sp.: characterization and application in starch degradation

Yan Li, Anan Li, Xue Long, Yuqing Cao, Aiyue Zhang, Jingang Gu, Rui Ma* and Guogang Zhao*

Table S1. *Hf*CGT Purification Table.

**Purification step**	**Total activity(U)**	**Total protein(mg)**	**Specific activity(U/mg)**	**Purification (fold)**	**Yield (%)**
Crude enzyme	120.7	80.4	1.5	1	100
Ni-NTA	58.4	5.7	10.3	6.9	48.4

Table S2. The LR-CD products from *Hf*CGT hydrolysis of soluble starch.

Compound	Molecular Weight (g/mol)	Observed *m/z* [M + H⁺]
$(C_6H_{10}O_5)_9$	1458.47	1459.48
$(C_6H_{10}O_5)_{10}$	1620.53	1621.54
$(C_6H_{10}O_5)_{11}$	1782.58	1783.59
$(C_6H_{10}O_5)_{12}$	1944.63	1945.64
$(C_6H_{10}O_5)_{13}$	2106.69	2107.69
$(C_6H_{10}O_5)_{14}$	2268.74	2269.75
$(C_6H_{10}O_5)_{15}$	2430.79	2431.80
$(C_6H_{10}O_5)_{16}$	2593.85	2594.85
$(C_6H_{10}O_5)_{17}$	2754.90	2755.91
$(C_6H_{10}O_5)_{18}$	2916.95	2917.96
$(C_6H_{10}O_5)_{19}$	3079.00	3080.23
$(C_6H_{10}O_5)_{20}$	3241.06	3242.08

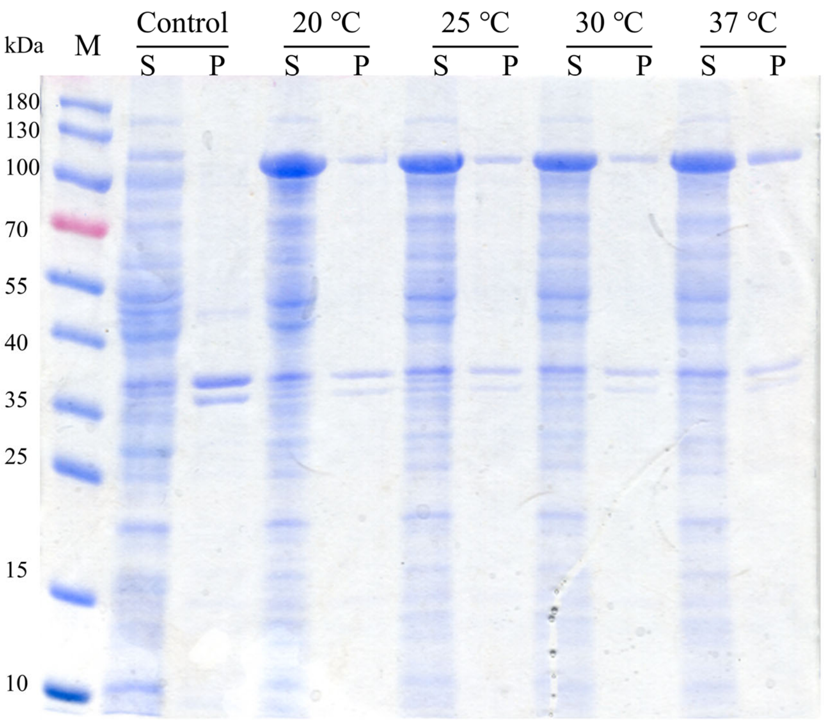

Figure S1 SDS-PAGE analysis of plasmid expression at different induction temperatures
Lane M:Protein Marker, Lane S: *Hf*CGT supernatant, Lane P: *Hf*CGT precipitate
20, 25, 30, 37 °C are the induction temperatures.
